# PrimerEvalPy: a tool for in-silico evaluation of primers for targeting the microbiome

**DOI:** 10.1186/s12859-024-05805-7

**Published:** 2024-05-14

**Authors:** Lara Vázquez-González, Alba Regueira-Iglesias, Carlos Balsa-Castro, Nicolás Vila-Blanco, Inmaculada Tomás, María J. Carreira

**Affiliations:** 1 Centro Singular de Investigación en Tecnoloxías Intelixentes (CiTIUS), Universidade de Santiago de Compostela, Rúa de Jenaro de la Fuente Domínguez, E15782 Santiago de Compostela, Spain; 2 Departamento de Electrónica e Computación, Escola Técnica Superior de Enxeñaría, Universidade de Santiago de Compostela, E15782 Santiago de Compostela, Spain; 3 Oral Sciences Research Group, Special Needs Unit, Department of Surgery and Medical Surgical Specialities, School of Medicine and Dentistry, Universidade de Santiago de Compostela, E15782 Santiago de Compostela, Spain; 4 Instituto de Investigación Sanitaria de Santiago de Compostela (IDIS), E15706 Santiago de Compostela, Spain

**Keywords:** Bioinformatics, Primer, 16S rRNA gene, Microbiome, Sequence analysis

## Abstract

**Background:**

The selection of primer pairs in sequencing-based research can greatly influence the results, highlighting the need for a tool capable of analysing their performance *in-silico* prior to the sequencing process. We therefore propose PrimerEvalPy, a Python-based package designed to test the performance of any primer or primer pair against any sequencing database. The package calculates a coverage metric and returns the amplicon sequences found, along with information such as their average start and end positions. It also allows the analysis of coverage for different taxonomic levels.

**Results:**

As a case study, PrimerEvalPy was used to test the most commonly used primers in the literature against two oral 16S rRNA gene databases containing bacteria and archaea. The results showed that the most commonly used primer pairs in the oral cavity did not match those with the highest coverage. The best performing primer pairs were found for the detection of oral bacteria and archaea.

**Conclusions:**

This demonstrates the importance of a coverage analysis tool such as PrimerEvalPy to find the best primer pairs for specific niches. The software is available under the MIT licence at https://gitlab.citius.usc.es/lara.vazquez/PrimerEvalPy.

**Supplementary Information:**

The online version contains supplementary material available at 10.1186/s12859-024-05805-7.

## Introduction

High-throughput amplicon sequencing has become a fundamental tool in modern microbiome analysis. Although the 16S rRNA gene remains the best known and most studied gene, mainly for the study of bacteria and archaea [1], other genes, such as 18S rRNA [2], provide valuable insights into microbial eukaryotes, including protozoa and fungi. In addition, the Internal Transcribed Spacer (ITS) gene [3] and the 23S rRNA gene [4], although less widely used than 16S rRNA, have proved useful in exploring the diversity of microbial communities, particularly in identifying specific archaea and bacteria.

These genes often have several conserved regions. In some cases, such as the 16S rRNA, there can be up to nine regions that serve as target sites for primer-based amplicon amplification. Primers can be designed to amplify adjacent or distant regions, or even both ends of the gene. The latter, as seen in the 16S rRNA gene, is particularly important when using new massive high-throughput sequencing platforms, such as PacBio [5].

There is also a wide range of sample types suitable for analysis, ranging from oceanic [6] and environmental [7] to human, animal and food [8]. Within each of these categories, different niches often have very different sequence compositions, requiring specialised analytical approaches.

In all of the above scenarios, there may be dozens or even hundreds of primers available. Some of these are called universal primers and allow the simultaneous study of several taxonomic groups. For example, in the case of the 16S rRNA gene, these universal primers allow the study of both bacteria and archaea. Alternatively, specific primers are designed to target particular taxonomic groups, focusing exclusively on either archaea or bacteria. Researchers can also go even deeper and use primers to study smaller taxonomic subsets, such as specific genera or phyla within different samples.

Similarly, the 18S rRNA gene offers universal primers [9], but also primers tailored for exclusive use in the study of fungi, protozoa, or algae [10]. For the study of fungal diversity using the ITS gene, several primer pairs are proposed for different regions (e.g., ITS1F/ITS2 and ITS3/ITS4) [11]. These primers, some universal and some specific, target different taxonomic groups, including ascomycetes, basidiomycetes, ectomycorrhizal, arbuscular mycorrhizal fungi, and others. In addition, specialised primers are available to target fungal pathogens, whether in environmental or clinical samples [12, 13].

In the scientific literature, primer pairs have been proposed for specific niches, such as the oral cavity [14], or for specific taxonomic groups in samples from oceanic environments, soil, and other sources [15]. However, it is noteworthy that primers originally designed for environmental samples have been used in very different contexts [16].

In conclusion, with the constant emergence of new primer proposals and the already large number of potential primer candidates, there is an urgent need for a versatile tool to test the performance of these primer pairs against specific sequence databases. This tool should allow researchers to assess their performance before embarking on wet lab experiments. In order to accommodate the wide range of sample types mentioned above, this tool must include the following features:

- Evaluation of multiple candidate primers, either individually or in pairs.
- Analysis on any sequence database.
- Optional inclusion of taxonomic information to assess coverage across different taxonomic levels.
- Analysis of all clades.
- Output of primer start and end positions within the sequence.
- Support for whole genome analysis.

When evaluating primer pairs, the tool should also allow users to set minimum and maximum amplicon length values before starting coverage analysis. This last feature will make it easier for users to select the most appropriate sequencing platform for their research needs. With this comprehensive set of features, researchers will have access to richer and more relevant information for selecting optimal primers or primer pairs tailored to their specific research objectives.

There are several works in the literature that analyse primers to assess their quality, such as EMBOSS [17], Metacoder [18], TestPrime [19] and PrimerTree [20]. However, none of them fulfil all of the above criteria.

Therefore, in this work, we present PrimerEvalPy - a versatile tool designed for the in-silico evaluation of primers or primer pairs against specific sequence databases provided by the user. The above features have been incorporated into PrimerEvalPy. In addition, users can seamlessly access genomes using our tool to retrieve them from the National Center for Biotechnology Information (NCBI) databases by specifying the appropriate identifiers. Alternatively, PrimerEvalPy allows for the direct analysis of sequences without the need for prior downloads from the NCBI.

To assess the capabilities of this in-silico tool, we performed tests using the most commonly used primer pairs for the 16S rRNA gene in oral cavity research [14]. We tested primers targeting bacteria, archaea, and both (universal primers). These tests were carried out analysing an oral bacterial sequence database proposed by Escapa et al. [21] and improved by our research group, who also developed an oral archaeal database [14].

While we focus our attention on evaluating PrimerEvalPy on the oral microbiome, which has a limited diversity [22], it is important to highlight that this tool has the capacity to work with multiple and diverse niches.

## Implementation

PrimerEvalPy has been developed in Python 3.9, using Biopython [23], a well-known bioinformatics package, to support the handling of sequencing data. Our tool can be used both from the command line as well as integrated into other Python projects. It is also compatible with Windows and Linux.

The package accepts two primary inputs: primer sequences and the gene or genome sequences against which the primers are to be evaluated.

PrimerEvalPy has two modules that provide the main functionality of the package. The first is the analyze_ip module, designed for the analysis of single primer sequences, while the second is the analyze_pp module, tailored for the analysis of primer pairs.

The primer sequences can be evaluated on DNA sequences of different origins, provided they are presented in a FASTA file format. By default, the package returns coverage calculations for all sequences within the provided file. In cases where an additional file containing the taxonomy of all sequences is provided, PrimerEvalPy extends its capabilities to compute coverage at different taxonomic levels and even for all possible clades.

The package also includes the download module, which retrieves DNA sequences, either genes or genomes, from the NCBI nucleotide database. If desired, this module can also be used to retrieve and save the taxonomy.

All in all, PrimerEvalPy returns the results of the coverage analysis in several files. For each primer analysed, a table is generated, containing mainly the coverage and the average start and end positions of the primer in the sequences. FASTA files containing the sequences found by the primer are also generated.

### Input file for target primers

The list of primers to be evaluated should be in the oligo file format used by Mothur [24]. This file format indicates whether a primer is a single primer (denoted by ‘forward’ or ‘reverse’) or a primer pair (denoted by ‘primer’). It includes their sequence(s) and optionally a name for identification.

It is important to note that PrimerEvalPy supports primers with degenerate bases as defined by the International Union of Pure and Applied Chemistry (IUPAC), which are treated accordingly during the analysis. However, no other transformation is applied to these sequences, so they must be presented in the correct direction for amplification.

### Input file for gene or genome sequences, and taxonomy

The genes and genomes against which the candidate primers are to be evaluated must be provided in FASTA formatted files. It is also possible to download them directly from the NCBI database using the PrimerEvalPy download module.

The taxonomy for each sequence can also be provided. This should be in a separate taxonomy file with the same name as the corresponding FASTA file. This contains one line per sequence, including its identifier (matching the one in the FASTA file), and the taxonomy itself, with each taxonomic level separated by semicolons. The user must specify the name for each taxonomic level to be read from the files, and all files must contain the same number of taxonomic levels.

### Primer coverage analysis procedure

**Fig. 1:**
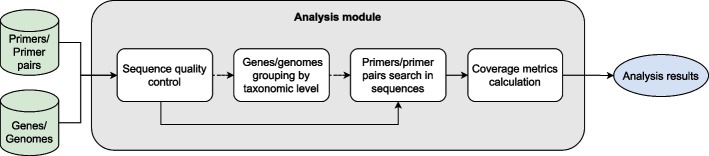
Block diagram of the analysis process for testing a primer or primer pair against a specific database. It consists of a sequence quality control step, followed by an optional sequence grouping step at the taxonomic level, then a primer search step within the sequences, and finally a coverage metrics calculation step

To calculate the primer coverage measurements, as well as other functionality, we follow a series of steps shown in Fig. 1, which will be explained in the following subsections.

#### Step 1: Sequence quality control

The first step in both the analyze_ip and analyze_pp modules is a quality check of the sequences provided. This quality check involves the identification of any degenerate nucleotides that could potentially affect the subsequent analysis.

During this process, the modules actively search for nucleotides beyond the four basic bases (A, C, G, and T). If a non-standard nucleotide is detected, such as U (Uracil) found in RNA, it is clearly marked. While these unwanted nucleotides are flagged for user awareness, it is up to the user to decide what to do with them.

This quality control procedure ensures that the input data meets the required quality standards before the analyses are performed. It allows users to make informed decisions about the inclusion or exclusion of sequences based on their quality.

#### Step 2: Sequence grouping by taxonomic level

By default, PrimerEvalPy does not specify a taxonomy level for grouping sequences. Therefore, each sequence is analysed individually and forms its own group. In this way, it is analysed whether a sequence is covered by the primer being evaluated.

**Fig. 2:**
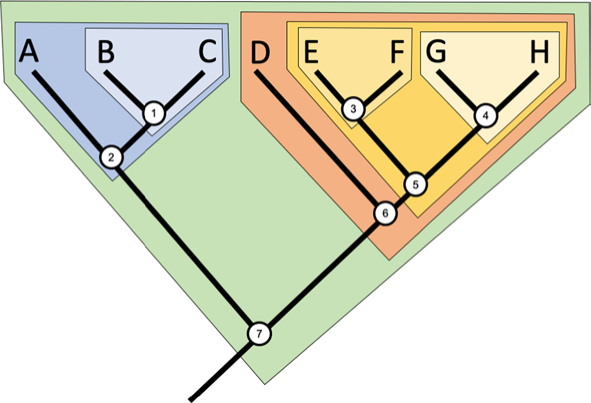
Example of a phylogenetic tree highlighting the clades, where node 5 represents the common ancestor of nodes 3 and 4, forming a clade that includes all three. Image by J.R. Hendricks [25] licensed under a Creative Commons Attribution-ShareAlike 4.0 International License

However, a key feature of PrimerEvalPy is that it supports coverage analysis at different taxonomic levels. It also allows grouping by all possible clades, i.e., groups formed by a common ancestor and all its descendants. This concept is illustrated in the phylogenetic tree in Fig. 2.

To evaluate sequences at different taxonomic levels, it is essential to have the appropriate taxonomy file and to specify the names of the taxonomic levels included. This allows the package to group the sequences at the taxonomic level desired by the user. When a taxonomic level is specified, PrimerEvalPy will search for all taxa within it, i.e., all groups of sequences that share the same taxonomic classification up to that level. The sequences from each taxon form an analysis group.

#### Steps 3 and 4: Primer search in sequences and assessment of coverage metrics

When expressed as a percentage, coverage represents the proportion of target sequences in a given dataset that can be effectively amplified by a specific primer or primer pair. It quantifies the primer’s efficiency in capturing and amplifying the genetic material of interest within the sample.

The primer sequences provided in the oligo file contain the four nucleobases A, C, G and T, but may also contain degenerate bases (IUPAC codes). We have therefore used regular expressions (regex) to search for the primers, either individually or in pairs, within the gene or genome sequences. These replace the degenerate bases with their possible corresponding nucleobases to ensure accurate matches within the sequences.

Furthermore, a maximum number of “mismatches” was allowed when searching for the primer within the sequence. To facilitate this, regex with fuzzy matching is used, meaning that some nucleotides in the sequence may not exactly match the corresponding nucleotides in the primer sequence. By default, no mismatches are allowed.

In addition, for primer pairs, the user can specify a minimum and maximum length of the amplified fragment between the forward and reverse primers.

Once the sequences amplified by the primer have been found and stored, coverage metrics are calculated. Primarily, the percentage of groups covered by the primer out of the total number of groups is calculated to determine the coverage of the primer. A group is considered to be covered if any of its sequences are found by the primer. If no taxonomic level was specified, which is the default approach, each sequence constitutes a group, so the coverage is the percentage of sequences covered by the primer. If a taxonomic level was specified, each group corresponds to a taxon. The most common is species level coverage, which is the percentage of species covered, that is, what percentage of species have at least one of their sequences amplified by the primer. There is also an option to obtain group coverage, which is the percentage of sequences within each group that are covered.

### Download complete genomes from NCBI

**Fig. 3:**
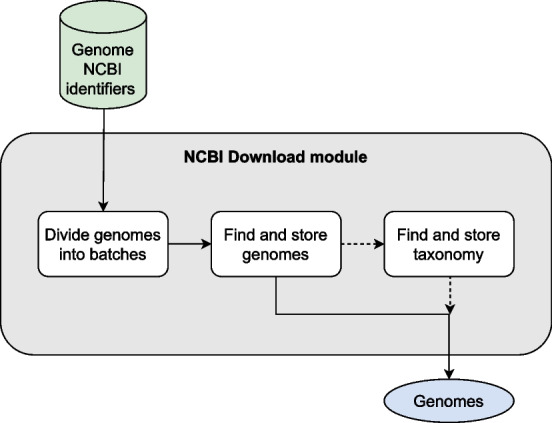
Block diagram of the steps of the NCBI download module. Here the genome identifiers are divided into batches to download the genome sequences from the NCBI step by step. Optionally, the taxonomy can also be retrieved and linked to the corresponding genomes

PrimerEvalPy includes a complementary module that allows users to download complete genomes or genes from the NCBI databases, as shown in Fig. 3. Although not a core feature, this option significantly enhances the capabilities of the tool and facilitates the analysis process.

Sequences are downloaded from the NCBI nucleotide database using the Entrez module of the Biopython package, which is a wrapper for the online search system of the same name provided by NCBI. To use this module effectively, users must use the accession identifiers used by NCBI. It also offers the option of downloading the relevant taxonomies, which enrich the dataset with essential contextual information.

## Results

As a practical case, PrimerEvalPy was used to test the most commonly used primers in the literature against two 16S rRNA gene oral databases containing bacteria and archaea. The article by Regueira et al. [14] provides a detailed analysis.

The bacterial dataset improved by our research group was the Escapa et al. [21] dataset, which contains a total of 223,143 amplicon sequence variants (ASVs) of FASTA-formatted 16S rRNA gene sequences, and a total of 769 oral bacterial species. In particular, sequences from the same hierarchy were simultaneously aligned using Clustal Omega against a set of Escherichia coli 16S rRNA gene sequences. This dataset is provided in the Supplementary information [see Additional file 1]. The archaeal dataset was generated by our research group from complete genomes of the human oral archaeal species from the NCBI nucleotide database. This included 2842 16S rRNA gene sequences and 196 archaeal species, and is provided in the Supplementary information [see Additional file 2].

A total of 456 individual primers were analysed with PrimerEvalPy at the variant and species level, including forward, reverse, and unknown primers. These are provided in the Supplementary information [see Additional file 3]. Of these, 356 targeted bacteria, 79 archaea, and 21 both (universal) according to the literature. However, we found that some primers at the species level covered a different domain than expected, as shown in Table 1. Many primers that were thought to cover only bacteria turned out to cover both bacteria and archaea. In addition, 26 were found to have no coverage at all in the oral cavity. We also observed that the primers with the best coverage identified in the study were not among those commonly described in the oral microbiome literature.

**Table 1:** Number of primers covering bacteria, archaea, both (universal) or none at the species level, comparing those described in the literature with those classified by PrimerEvalPy

	Bacteria	Archaea	Universal	None
Literature	356	79	21	0
PrimerEvalPy	200	64	166	26

Next, the primers with coverage at the species level $\ge 75\%$ (148 bacterial and 65 archaeal primers) were selected to form valid primer pairs. All possible combinations of the forward and reverse primers were identified, resulting in a total of 4,638 primer pairs. These were again evaluated to find the best ones for the detection of oral bacteria and archaea.

It was discovered that the primer pairs with the highest coverage, as proposed in the literature, did not cover many oral species that were covered by other primer pairs constructed and evaluated in this study. Additionally, the primer pairs identified as the best by PrimerEvalPy did not align with those found to be the best in the literature.

## Discussion

PrimerEvalPy allows for the evaluation of primers and primer pairs using their coverage as a measure of their quality. Although there are several works in the literature that analyse primers in a similar way, they have disadvantages ranging from availability in Python to limitations in the analysis itself. Only PrimerEvalPy includes analysis of individual primers, analysis of primer pairs and analysis for different taxonomic ranks, i.e., taxonomic levels, on any database. Table 2 shows a comparison of the functionalities of PrimerEvalPy with other packages.

**Table 2:** Comparison of PrimerEvalPy with other tools

Functionality	EMBOSS [17]	Metacoder [18]	TestPrime [19]	PrimerTree [20]	PrimerEvalPy (ours)
Individual primer analysis					$\checkmark$
Average start & end positions					$\checkmark$
Average amplicon length					$\checkmark$
Coverage score			$\checkmark$		$\checkmark$
Taxonomic level analysis		$\checkmark$			$\checkmark$
Available in Python	$\checkmark$				$\checkmark$
Multiple primer analysis	$\checkmark$	$\checkmark$			$\checkmark$
Custom database	$\checkmark$	$\checkmark$			$\checkmark$
Primer pair analysis	$\checkmark$	$\checkmark$	$\checkmark$	$\checkmark$	$\checkmark$
Mismatches allowance	$\checkmark$	$\checkmark$	$\checkmark$	$\checkmark$	$\checkmark$

One such tool is the European Molecular Biology Open Software Suite [17], known as EMBOSS. This is only available for UNIX systems via the command line. It allows you to analyse a pair of primers on one or more sequences, taking into account mismatches. There are many tools that use EMBOSS, such as the Emboss module in Biopython [23]. This is a wrapper for the EMBOSS toolkit and does not add any functionality. Like EMBOSS, it does not support individual primer analysis, nor does it provide coverage information that needs to be calculated. It also does not include the analysis for different taxonomic levels.

Another tool that uses EMBOSS is the R package Metacoder [18]. It allows for primer pair analysis using EMBOSS, but has been extended with additional functionality. Metacoder adds the analysis for different taxonomic levels and provides coverage measurements. However, it is only available for R, not for Python, and like EMBOSS it does not support individual primer analysis. It provides the start and end positions of each amplicon in the sequences, as well as their length, but not the average. Also, as it is based on EMBOSS, it is not available for Windows.

Apart from the tools using the EMBOSS suite, there is a web tool called TestPrime [19] which allows the analysis of one primer pair at a time only on the proposed Silva databases (PCR in silico). Like the others, it allows the analysis of primer pairs with mismatches on the primers and gives coverage information. However, it is only available as a web tool, not for Python or R, and does not allow individual primer analysis. It provides the amplicon length, but not its average or the start and end positions. Also, primers cannot be analysed in any database, there are only two to choose from.

Finally, another analysed tool was PrimerTree [20], an R package that allows the analysis of a primer pair on a specific NCBI database using Clustal Omega. This tool analyses one primer pair at a time, allowing for mismatches on the primers, and returns the number of alignments performed between the primer pair and the sequences. However, it can only be applied to the specified ecology dataset and cannot be used to analyse other datasets. It provides the start and end positions of each amplicon in the sequences, as well as its length, but does not provide the averages of the above values. In addition, it does not provide coverage measurements, does not support analysis of individual primers, nor analysis for different taxonomic levels.

PrimerEvalPy is the only tool that has all the desired features, as shown in Table 2. Unlike all the other tools, it is the only one that allows the analysis of individual primers and calculates the average start and end positions of the primer in the sequences.

As validation, PrimerEvalPy was compared to Metacoder, the tool with most functionality from those available in the literature. Given that Metacoder does not include individual primer analysis, only three of the best primer pairs targeting bacteria and three of the best targeting archaea (according to PrimerEvalPy) were evaluated and compared against the bacteria database and archaea database, respectively. The same species level coverage was obtained for each primer pair with both tools.

## Conclusion

The PrimerEvalPy package allows the analysis of individual primers or primer pairs. Several measures are returned to help make an informed decision, and there are several options to fine-tune the analysis. Analysis is also available at different taxonomic levels, allowing researchers to explore the suitability of primers for specific ranks in the niche.

We believe that this tool can be of great value to researchers wishing to study niche diversity using high-throughput amplicon sequencing techniques. Users can efficiently compare large numbers of primers in an economical and rapid manner, thereby reducing the number of primers that need to be evaluated in the laboratory. It also facilitates the seamless modification of primers derived from existing literature, allowing subsequent evaluation for potential improvements.

The results obtained in the case study demonstrated the need for such a tool. They showed that some of the primer pairs with the highest coverage suggested by the literature did not match the best found with PrimerEvalPy. Furthermore, some of the primers studied did not have coverage in the oral cavity, highlighting the importance of a prior study focusing on the target niche.

Although there are many tools that address this problem of primer coverage analysis, many of them have several of the limitations mentioned above. With PrimerEvalPy, we aim to overcome these limitations and provide a useful and practical tool.

In conclusion, PrimerEvalPy is a fundamental tool that allows in-silico primer analysis prior to any sequencing process, thus contributing to improve the quality and reliability of the microbial diversity results of any ecosystem.

## Availability and requirements

- **Project name**: PrimerEvalPy
- **Project home page**: https://gitlab.citius.usc.es/lara.vazquez/PrimerEvalPy
- **Operating system(s)**: e.g. Platform independent
- **Programming language**: e.g. Python
- **Other requirements**: Python 3.9 or higher
- **License**: MIT License
- **Any restrictions to use by non-academics**: None

### Supplementary information

**Additional file 1: Oral-bacteria database of the 16S rRNA gene sequences which was used for the coverage analysis.**

**Additional file 2: Oral-archaea database of the 16S rRNA gene sequences which was used for the coverage analysis.**

**Additional file 3: Forward and reverse 16S rRNA gene primers that were evaluated in the study.**

## Data Availability

The datasets used or analysed in this study were obtained from the Regueira et al. [14] article and are available in this manuscript as Supplementary information.
